# Fecal Carriage of ESBL-Producing *E. coli* and *K. pneumoniae* in Children in Guinea-Bissau: A Hospital-Based Cross-Sectional Study

**DOI:** 10.1371/journal.pone.0051981

**Published:** 2012-12-20

**Authors:** Joakim Isendahl, Agata Turlej-Rogacka, Cristovão Manjuba, Amabelia Rodrigues, Christian G. Giske, Pontus Nauclér

**Affiliations:** 1 Department of Microbiology, Tumor and Cell Biology, Karolinska Institutet, Stockholm, Sweden; 2 Department of Clinical Microbiology, Karolinska Institutet, Stockholm, Sweden; 3 Department of Pediatrics, Hospital Nacional Simão Mendes, Bissau, Guinea-Bissau; 4 Instituto Nacional da Saúde Pública, Bissau, Guinea-Bissau; 5 Department of Infectious Diseases, Karolinska University Hospital, Stockholm, Sweden; University of Calgary, Canada

## Abstract

**Background:**

In recent years, the world has seen a surge in extended-spectrum β-lactamase (ESBL)-producing bacteria. However, data on the dissemination of ESBL-producing Enterobacteriaceae in the community from systematically enrolled study subjects in Africa remains limited. To determine the prevalence, phenotypic resistance patterns and genetic characteristics of ESBL-producing *E. coli* and *K. pneumoniae* in fecal carriage and to analyze associated risk factors in children attending a pediatric emergency department in Guinea-Bissau.

**Methodology/Principal Findings:**

From June to September 2010, children <5 years of age with fever or tachycardia attending a pediatric emergency ward during the day was screened for ESBL carriage in feces. Socio-demographic and health seeking behavior data was collected. Antibiotic susceptibility was tested with VITEK2 and EUCAST disk diffusion method, molecular characterization of ESBL-encoding genes was performed with multiplex PCR and clonal relatedness was established by automated rep-PCR. Of 408 enrolled children 133 (32.6%) were ESBL carriers. In total, 83 *E. coli* and 91 *K. pneumoniae* ESBL-producing isolates were obtained. Nearly all isolates were multidrug-resistant. Co-resistance to ciprofloxacin, trimethoprim-sulfamethoxazole and aminoglycosides was common. Of the isolates, 38.5% were co-resistant to these classes plus extended-spectrum cephalosporins, which infers resistance to all easily available antibiotic agents for treatment of gram-negative sepsis in Guinea-Bissau. The predominant resistance-encoding gene subgroup was *bla*_CTX-M-1_ and epidemiologic typing showed that the bacterial ESBL population was highly diverse both for *E. coli* and *K. pneumoniae*. Bed sharing with another child <5 years of age was a risk factor for ESBL carriage, indicating crowding as a potential risk factor for transmission of ESBL-producing bacteria.

**Conclusions/Significance:**

Prevalence of ESBL-producing bacteria in this population was high and clonally diverse. This is alarming considering the limited diagnostic and treatment possibilities in Guinea-Bissau and other resource-poor countries.

## Introduction

During the past few decades, ever-increasing use of antibiotic agents has led to selective pressure in favor of bacteria that have acquired resistance enzymes [1]. *Extended spectrum β-lactamases* (ESBLs) are acquired enzymes that hydrolyze extended-spectrum cephalosporins and which by the classical definition are inhibited by clavulanic acid [2]. ESBLs are currently spreading rapidly amongst Enterobacteriaceae, largely due to genes located on plasmids that can disseminate across species barriers [3], [4]. The *bla*_CTX-M_ genes are located on such plasmids and specifically the presence of the *bla*_CTX-M-15_ gene has been linked to the ongoing spread of ESBLs globally [5]. Such dissemination of CTX-M-producing Enterobacteriaceae strains is an emerging public health concern [6]. Plasmid-mediated transfer of ESBLs also results in increasing resistance to non-β-lactams such as quinolones, since plasmids can harbour genes that confer resistance to multiple antibiotic groups [1], [7].

*Escherichia coli* (*E. coli*) and *Klebsiella pneumoniae* (*K. pneumoniae*) are common species of Enterobacteriaceae that both have pathogenic potential and that frequently incorporate ESBL-encoding genes [8]. The Infectious Diseases Society of America has listed them as two out of six pathogens for which new drugs are urgently needed in order to combat resistance development [9]. A meta-analysis reported that infections with ESBL-producing bacteria are associated with nearly twice the mortality compared to that of non-ESBL producers [10]. Even in the absence of infection, colonization with ESBL-producing bacteria is a reason for concern. Carriage of resistant commensal Enterobacteriaceae strains in the normal gut flora may serve as a reservoir of resistance genes that subsequently may be acquired by strains that cause infection [3].

Studies from different countries report varying prevalence of colonization [11]–[21] and data on the spread of ESBL-producing bacteria from Sub-Saharan Africa is needed to assess the extent of this emerging health threat in resource-poor settings. There are sporadic reports about the prevalence of ESBL-producing bacteria in clinical isolates in Africa but there are few studies that have systematically collected data on the prevalence of colonization with these pathogens [17]–[19].

Guinea-Bissau is a resource-poor country of approximately one and a half million inhabitants [22] with limited means to meet the challenges of multi-drug resistant pathogens. This study aimed to determine the prevalence, phenotypic resistance patterns and genetic characteristics of ESBL-producing *E. coli* and *K. pneumoniae* in fecal carriage in children attending a pediatric emergency department in Guinea-Bissau. It also sought to identify risk factors for gut colonization with ESBL-producing clones related to socio-demographic features and recent health status.

## Methods

### Study Population

This study was performed at the Pediatric Emergency Department at the National Hospital Simão Mendes, which is the only pediatric clinic in Bissau, the capital of Guinea-Bissau. It was part of a study aiming to assess the burden of antibiotic resistance in children with sepsis. The population of the capital was 423 000 in 2009 and 41.2% of the population in the country was aged 0–14 years [22]. Most urban dwellers rely on the hospital for medical care and Hospital Simão Mendes also serves as referral hospital for primary care facilities and regional hospitals in the country.

Two research nurses approached the guardians of children under five years of age presenting with tachycardia (<1 year ≥160 and 1–5 years ≥120 beats per minute) and/or fever (≥38°C), to inform and ask for consent of their child’s participation in the study. Enrolment was carried out from June 8^th^ to September 22^nd^ 2010 on weekdays between 9 am and 5 pm. The guardians of all participants were subjected to a standardized interview from which socio-demographic data and data on recent health seeking behavior and medication was obtained. The number of guardians that denied participation is unknown but according to the study nurses very few guardians did not consent. Study supervisors visited the study site daily and the enrollment frequency of children that met the inclusion criteria was high.

### Fecal Cultures and Antibiotic Resistance Testing

Each participant was sampled with a rectal swab prior to initiation of any treatment unless the procedure conferred a risk of delaying the initiation of treatment. The research nurses had been trained to insert the swab approximately one inch into the rectum and to thereafter gently rotate the swab. The fecal swabs were then put in a 114C.USE transport medium (Copan Italia S.p.A, Brescia, Italy) and stored in a refrigerator overnight. The next morning they were transported to and cultured at the Bacteriology Department at the National Public Health Laboratory of Guinea-Bissau. Samples were cultured on an in-house cysteine-lactose-electrolyte deficient medium and on ChromID ESBL (bioMérieux, Marcy l’Etoile, France), a commercial ESBL-selective chromogenic agar medium [23]. Unique colony morphologies growing on the selective agar were frozen in –20°C in a freezing medium for sensitive bacteria used by and manufactured at the Department of Clinical Microbiology at Karolinska University Hospital in Stockholm, Sweden. At the end of the study period all samples were transported on dry ice to the Department of Clinical Microbiology at Karolinska University Hospital in Stockholm, Sweden where they were frozen in –70°C before further phenotypic and molecular analyses.

At the laboratory in Sweden, all frozen strains were re-cultured on the ChromID ESBL-selective medium. Biochemical species identification and confirmation of ESBL-producing isolates was carried out using the semi-automated VITEK2 system (bioMérieux, Marcy l’Etoile, France) [24]. Susceptibility to cefotaxime, ceftazidime, gentamicin, tobramycin, tigecycline and amoxicillin-clavulanic acid was tested with the VITEK2 system and susceptibility to trimethoprim-sulfamethoxazole, piperacillin-tazobactam, ciprofloxacin and meropenem was tested with the antibiotic disc diffusion method (Oxoid, Basingstoke, UK) on Mueller-Hinton agar (Oxoid, Basingstoke, UK). Isolates were classified as susceptible or resistant based on recommended minimum inhibitory concentration and zone diameter breakpoints from the European Committee on Antimicrobial Susceptibility Testing [25]. In the analyses intermediately resistant and resistant isolates were classified as non-susceptible.

### Genetic Characteristics of ESBL-Producing Isolates

All confirmed ESBL-producing isolates were subjected to molecular testing to detect ESBL-encoding genes and phylogenetic groups. A multiplex, real-time TaqMan PCR assay was used to identify and distinguish *bla*_CTX-M_ genes into the four phylogenetic subgroups 1, 2, 9 and 8/25 as described by Birkett et al. [26]. Isolates negative for *bla*_CTX-M_ were analyzed for carbapenemase, AmpC, ESBL *bla*_TEM_ and *bla*_SHV_ genes with the commercial Check-MDR CT101 PCR assay (Check-Points, Wageningen, The Netherlands) [27].

The genetic relatedness of the isolates was investigated with the DiversiLab (DL) system (bioMérieux), a semi-automated repetitive-sequence PCR-based typing system [28]. A unique DL type was assigned to *K. pneumoniae* isolates clustering at ≥93% and *E. coli* isolates clustering at ≥95% similarity, in accordance with a previous study on the discriminatory capacity of the DL method [28]. Non-clustering isolates were labeled singletons. The genetic relatedness of isolates as determined by DL analysis was illustrated utilizing the Minimal Spanning Tree algorithm in Bionumerics (Applied Maths, NV St-Martens-Latem, Belgium). In order to identify isolates from Guinea-Bissau that belong to some important clonal lineages, DL analysis results of ESBL-producing isolates from Sweden previously shown to belong to *E. coli* multi-locus sequence type (MLST) 131 and *K. pneumoniae* STs 11, 14, 15 and 258 [28] were added to the DL software. They were analyzed together with the results of the DL analysis of the Guinea-Bissau isolates to assess the level of phylogenetic similarity.

### Ethics

Written informed consent was obtained or, if the parent or guardian was illiterate, a fingerprint served as proof of consent. The study was approved by the Guinea-Bissau Government Ethics Committee and the Regional Ethical Review Board in Stockholm, Sweden.

### Statistical Methods

The database was screened for outlying numerical observations and crosschecked before analysis. The Pearson chi-squared test and Fischer’s exact test were used as appropriate for categorical data and Student’s t-test was used for continuous data in the analyses of socio-demographic and health-status related factors associated with carriage of ESBL-producing *E. coli* or *K. pneumoniae*. Two-sided p-values ≤0.05 were considered statistically significant. All statistical calculations were performed with Stata version 12 (StataCorp LP, College Station, USA).

## Results

### Colonization Prevalence and Phenotypic Resistance Characteristics

In total, 447 children had a fecal sample taken. Out of these 29 were retrospectively excluded from the study since they had been misclassified as being part of the study group despite not fulfilling the inclusion criteria of fever and/or tachycardia. Furthermore, one child who was >5 years of age was excluded. Nine children were excluded since there was a lack of freezing media during two weeks in July. Hence, 408 patients were included in the analyses of which 133 (32.6%) were carriers of at least one ESBL-producing *E. coli* or *K. pneumoniae* strain. Simultaneous carriage of two strains was detected in 33 samples (8.1%) and carriage of three strains was present in another four samples (1.0%). Among the 174 detected ESBL-expressing isolates 83 were *E. coli* and 91 were *K. pneumoniae.* Quinolone resistance was common in *E. coli* with 81.9% of the isolates being resistant, but somewhat less frequent in *K. pneumoniae* with 48.4% of the isolates being resistant (Table 1). Resistance to aminoglycosides was high with 43.4% and 71.1% of *E. coli* isolates and 93.4% and 94.5% of *K. pneumoniae* isolates being resistant to gentamicin and tobramycin, respectively. Resistance to trimethoprim-sulfamethoxazole was high for both *E. coli* and *K. pneumoniae*, 94.0% and 91.2% respectively. Co-resistance to these three antibiotic classes was observed in 38.5% of the isolates. All isolates remained susceptible to carbapenems.

**Table 1:** Antimicrobial Non-Susceptibility of ESBL-Producing *E. coli* and *K. pneumoniae* in Guinea-Bissau^1^.

Antimicrobial agent		*E. coli*	*K. pneumoniae*
		% (n = 83)	% (n = 91)
***Ceftazidime***		94.0	97.8
***Cefotaxime***		98.7	97.8
***Gentamicin***		43.4	93.4
***Tobramycin***		71.1	94.5
***Trimethoprim-sulfamethoxazole***		94.0	91.2
***Meropenem***		0.0	0.0
***Ciprofloxacin***		81.9	48.4
***Piperacillin-tazobactam***		55.4	92.3
***Tigecycline***		0.0	2.2
***Amoxicillin-clavulanic acid***		62.7	77.0
**Multi-drug non-susceptible** 2		97.6	100
**Non-susceptible to all easily available antibiotics in Bissau** 3		33.7	42.9

1 Non-susceptibility of isolate was defined as intermediate (I) or resistant (R) to respective antibiotic agent.

2 Non-susceptibility of isolate to three or more categories of antimicrobial agents as proposed by Magiorakos et al. [30].

3 Available antimicrobials for common infections caused by gram-negative bacteria in Guinea-Bissau at the time of the study were trimethoprim-sulfamethoxazole, ciprofloxacin, gentamicin and ceftriaxone.

### Genetic Characteristics

PCR screening for resistance genes yielded that *bla*_CTX-M-1_ was the predominating β-lactamase enzyme group. In total, 165 isolates (94.8%) belonged to this phylogenetic group (96.4% of *E. coli* and 93.4% of *K. pneumoniae*). Four of these isolates (2.3%) were positive both for the *bla*_CTX-M-1_ and the *bla*_CTX-M-9_ phylogroup. Three isolates (1.7%) were positive for *bla*_CTX-M-9_, one isolate was positive for *bla*_CTX-M-8/25_ and one for *bla*_CTX-M-2_ genes. Four isolates expressed ESBL *bla*_SHV_ gene variants.

**Figure 1:**
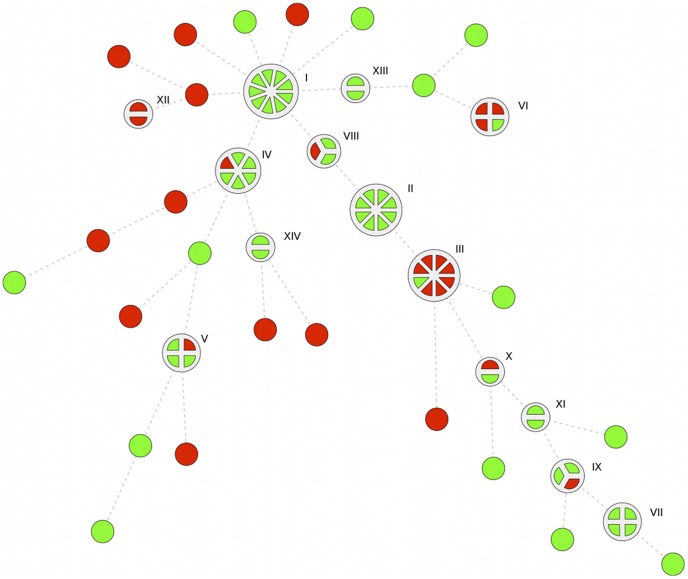
Minimal Spanning Tree of *E. coli* Isolates in Fecal Carriage in Children in Guinea-Bissau. Full legend: The tree maps the relatedness of *E. coli* isolates. Isolates ≥95% related in DiversiLab analysis are grouped in a pie where each slice represents one isolate in the cluster. Each cluster was assigned a Roman numeral. Red indicates resistance to gentamicin, ciprofloxacin and trimethoprim-sulfamethoxazole, which at the time of the study were all easily available antibiotics except cephalosporin to treat gram-negative bacterial infections in Guinea-Bissau. Green indicates susceptibility to at least one of the mentioned agents.

**Figure 2:**
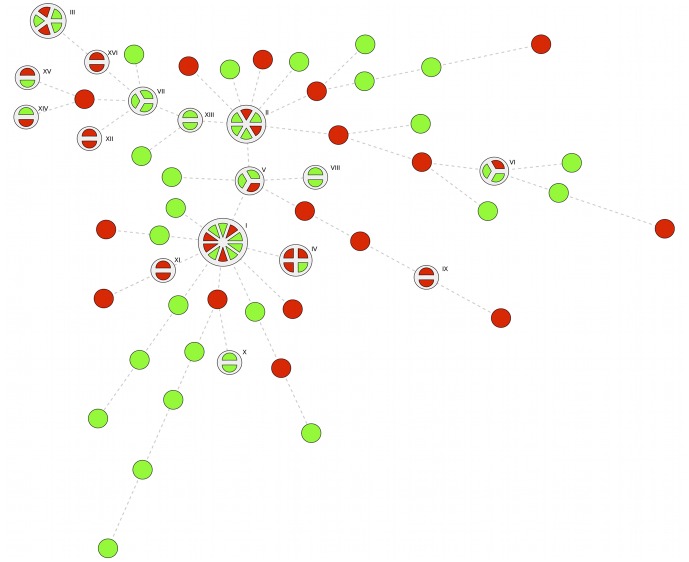
Minimal Spanning Tree of *K. pneumoniae* Isolates in Fecal Carriage in Children in Guinea-Bissau. Full legend: The tree maps the relatedness of *K. pneumoniae* isolates. Isolates ≥93% related in DiversiLab analysis are grouped in a pie where each slice represents one isolate in the cluster. Each cluster was assigned a Roman numeral. Red indicates resistance to gentamicin, ciprofloxacin and trimethoprim-sulfamethoxazole, which at the time of the study were all easily available antibiotics except cephalosporin to treat gram-negative bacterial infections in Guinea-Bissau. Green indicates susceptibility to at least one of the mentioned agents.

Genetic fingerprinting rendered 14 DL types for *E. coli*; the most prevalent containing nine, eight, eight and six isolates, respectively (Figure 1). Three *E. coli* DL types contained four isolates, two contained three isolates, five contained two isolates and 24 isolates were singletons. For *K. pneumoniae* 16 DL types were found, the most prevalent consisting of ten, six, five and four isolates, respectively (Figure 2). Three DL types contained three isolates, nine types contained two isolates and 39 isolates were singletons.

In an analysis where *E. coli* isolates from Guinea-Bissau were compared to isolates previously determined to belong to *E. coli* ST131, only three isolates from Guinea-Bissau clustered with the ST131 isolates. In a similar comparison of the epidemic *K. pneumoniae* clones ST 11, 14, 15 and 258 and the isolates from Guinea-Bissau, none of the isolates clustered.

### Risk Factors for ESBL Carriage

**Figure 3:**
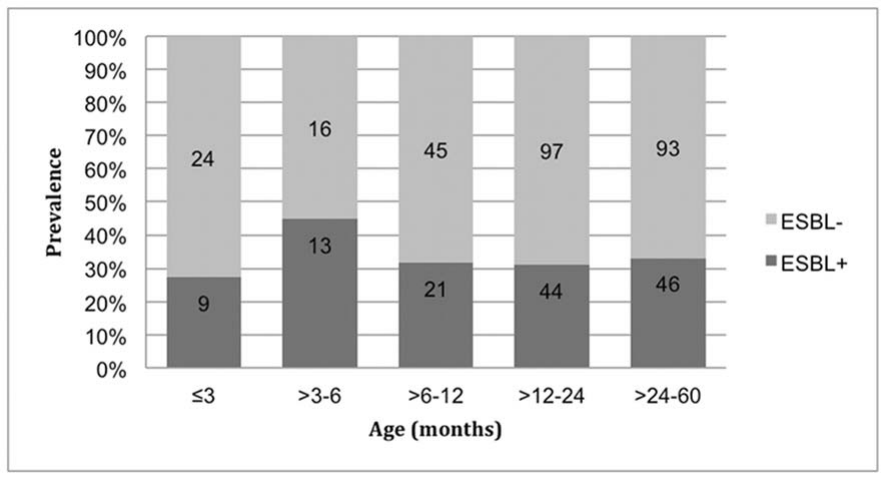
Carriage Prevalence of ESBL-Producing *E. coli* and *K. pneumoniae* According to Age. Full legend: The ESBL carriage prevalence did not vary depending on age. Absolute numbers are presented within bars.

Out of the 408 children included in the analyses 168 (41.1%) were female. (Table 2). The mean age of the study population was 1.71 years. There was no association between age and colonization with ESBL-producing *E. coli* or *K. pneumoniae* (p-value 0.71 and even in the youngest age group of 0–3 months 27% of the children were colonized) (Figure 3). Sharing bed with one or more children under the age of five was associated with increased risk of carriage (p = 0.04) of ESBL-producing *E. coli* or *K. pneumoniae* (Table 2). There was no association with previous usage of antibiotics or recent hospitalization but the reported antibiotic consumption and hospitalization was low in this population.

**Table 2:** Study Population Characteristics and Risk Factors for Colonization with ESBL-Producing Bacteria.

	Total	ESBL+	ESBL-		P-value
	n = 408 (%)	n = 133 (%)	n = 275 (%)		
**Gender (male)**	240 (58.8)	76 (57.1)	164 (59.6)		0.63
**Mean age (years)**	1.70	1.69	1.71		0.86
**Mean weight (kg)**	9.46	9.52	9.43		0.78
**MUAC** 1					
<115 mm	14 (3.4)	4 (3.0)	10 (3.6)		0.15^2^
115-<125 mm	29 (7.1)	12 (9.0)	17 (6.2)		
125-<135 mm	50 (12.3)	20 (15.0)	30 (10.9)		
≥135	253 (62.0)	75 (56.4)	178 (64.7)		
Data missing	62 (15.2)	22 (16.5)	40 (14.5)		
**Breastfeeding** 3					
Breastfed (all children)	189 (46.3)	55 (41.4)	134 (48.7)		0.19
Not breastfed (all children)	210 (51.5)	74 (55.6)	136 (49.5)		
Breastfed (children <1 year of age)	113 (27.7)	36 (27.1)	77 (28.0)		0.15
Not breastfed (children <1 year of age)	9 (2.2)	5 (3.8)	4 (1.5)		
Data missing	9 (2.2)	4 (3.0)	5 (1.8)		
**Bedsharing** 4					
Yes	51 (12.5)	23 (17.3)	28 (10.2)		0.04
No	351 (86.0)	107 (80.5)	244 (88.7)		
Data missing	6 (1.5)	3 (2.3)	3 (1.1)		
**Children in household** 5					
≥2	143 (35.1)	47 (35.3)	96 (34.9)		0.91
1	260 (63.7)	84 (63.2)	176 (64.0)		
Data missing	5 (1.2)	2 (1.5)	3 (1.1)		
**Started antibiotic treatment** 6					
Antibiotic	61 (15.0)	25 (18.8)	36 (13.1)	0.14	
No antibiotic	344 (84.3)	108 (81.2)	236 (85.8)		
Data missing	3 (0.7)		3 (1.1)		
**Antibiotic last month** 7					
No antibiotic	363 (89.0)	113 (85.0)	250 (90.9)		0.07
Antibiotic	10 (2.5)	6 (4.5)	4 (1.5)		
Data missing	35 (8.6)	14 (10.5)	21 (7.6)		
**Hospitalization last month** 8					
Hospitalized	6 (1.5)	3 (2.3)	3 (1.1)		0.40
Not hospitalized	352 (86.3)	116 (87.2)	236 (85.8)		
Data missing	50 (12.3)	14 (10.5)	36 (13.1)		

1 Mid-upper arm circumference at time of enrolment examined on children ≥6 months of age.

2 Test for linear trend.

3 Child breastfed at time of enrolment.

4 Bedsharing with another child <5 years of age.

5 Number of children <5 years of age living in the same household.

6 Antibiotic treatment initiated prior to presentation at emergency ward.

7 Reported antibiotic usage during the month prior to study enrolment (excluding antibiotic usage for current disease).

8 Child hospitalized ≥1 day during the month prior to enrolment.

## Discussion

In order to investigate the molecular epidemiology of carriage with ESBL-producing Enterobacteriaceae we systematically enrolled more than 400 children attending the pediatric emergency department at the national hospital in Guinea-Bissau. We report that 32.6% of the children were carriers of ESBL-producing *E. coli* or *K. pneumoniae.* The isolates were clonally diverse and 94.8% possessed *bla*_CTX-M-1_ genes that include the common *bla*_CTX-M-15_ gene [11]. Albeit this was a hospital-based study, the samples were collected directly upon presentation to the emergency ward and few study participants had a reported history of hospitalization or previous antibiotic usage. This indicates that the majority of ESBL-producing isolates were community-acquired.

There is limited data on the carriage prevalence of community-acquired ESBL-producing Enterobacteriaceae from Africa [17]–[19], [21]. The carrier prevalence in our study was higher than but comparable to a similar study in Madagascar that reported a prevalence of 21.1% among children admitted to hospital [17]. It has also been reported from Madagascar that 10.1% of 484 attendants to three health centers were ESBL carriers [18]. Among 55 acutely malnourished children with a medical condition admitted to a renutrition center in Maradi, Niger, 17 were ESBL carriers (30.9%) [19]. Recently in Cairo, Egypt a 63.3% carriage prevalence of ESBL-producing bacteria was reported in stool samples from 632 individuals attending a hospital clinic [20]. These data indicate that dissemination of ESBL-producing Enterobacteriaceae is substantial in the community in many parts of Africa.

In our study the prevalence was high in all age groups, also among the youngest where 27% were carriers in the ages 0–3 months. This indicates that colonization with ESBL-producing bacteria often occurs early in life in this population.

Our study reports that children who share bed have an increased risk of being colonized with ESBL-producing clones, indicating that close contact and crowding might be important for the spread of ESBLs. Other studies have identified antibiotic usage and previous contact with health care as risk factors for ESBL colonization or infection with ESBL-producing bacteria [17], [29]–[31]. We did not observe an increased risk of ESBL carriage among study participants with a history of health care contact or antibiotic usage. However the reported frequencies of these exposures were low in the study population, possibly due to misclassification.

The vast majority of our ESBL samples belonged to the *bla*_CTX-M-1_ gene group that includes *bla*_CTX-M-15_, the dominating gene group in fecal carriage studies from Madagascar, Niger, Cameroon, Tanzania and South Africa [18], [19], [21], [32], [33], highlighting the importance of these genes in the ongoing global dissemination of ESBLs [30].

Dissemination of the highly virulent clone ST 131 has been pointed out as responsible for clinical infections with ESBL-producing *E. coli* [34] and parallels the global dissemination of the *bla*_CTX-M-15_ gene [30]. However, in our study only three isolates from Guinea-Bissau clustered with previously identified ST 131 isolates, providing no evidence that *E. coli* ST 131 is a main contributor to the spread of ESBL-producing *E. coli* in Guinea-Bissau. Rather, the DL analyses showed that there was great genetic diversity among the samples, indicating that a multitude of circulating clones have acquired resistance genes. Similar observations have been made in Tanzania [32] and Madagascar [18], underscoring the effectiveness of plasmid exchange of genetic material within Enterobacteriaceae species.

Multidrug-resistance (MDR) was very common, with 172 out of 174 ESBL-producing isolates being classified as MDR according to the definition proposed by Magiorakos et al. [35]. Quinolone resistance was high in ESBL-producing *E. coli* (81.9%) and *K. pneumoniae* (48.4%) isolates. This can be compared to reports from other West African settings with 52–67% resistance prevalence reported from Accra, Ghana [36] and 74% in samples from patients with urinary tract infections in Dakar, Senegal [37]. In our study 38.5% of the ESBL-producing isolates were co-resistant to ciprofloxacin, gentamicin and trimethoprim-sulfamethoxazole. This is alarming since apart from cephalosporins these are the only easily available antibiotic agents to treat infections with gram-negative bacteria in Guinea-Bissau. Expensive second line antibiotics such as carbapenems largely remain unavailable.

Our study has some limitations. First, the hospital-based design means that our estimates are not generalizable to the community as there might be selection bias in the inclusion of patients. Nevertheless, a history of hospitalization and previous antibiotic usage was infrequently reported among included children. Second, it is possible that there was an underreporting of antibiotic usage during the last month due to antibiotics being dispensed without prescription in the local markets and due to lack of knowledge of medication classes. The assessment of antibiotic consumption as a risk factor for ESBL carriage therefore needs to be interpreted with caution. Third, analyses for acquired AmpC, ESBL *bla*_TEM_ and *bla*_SHV_ genes were only performed on *bla*_CTX-M_-negative isolates and hence there might be an underreporting of these genes.

In conclusion, we report a high prevalence of ESBL carriage with multiple clones in children presenting at a pediatric department in Guinea-Bissau. This observation underscores the need to improve microbiological diagnostic facilities and antibiotic resistance surveillance in resource-poor settings to be able to promptly revise antibiotic regimens to tackle resistance development. Large-scale, population-based epidemiological studies in resource-poor settings are warranted to better understand dissemination patterns and societal impact of ESBL-producing organisms.
